# Designated functional microcapsules loaded with green synthesis selenium nanorods and probiotics for enhancing stirred yogurt

**DOI:** 10.1038/s41598-022-18781-w

**Published:** 2022-08-30

**Authors:** Hoda S. El-Sayed, Samah M. El-Sayed, Ahmed M. Youssef

**Affiliations:** 1 Dairy Science Department, National Research Centre, 33 El Bohouth St. (former El Tahrir St.), Dokki, Giza, 12622 Egypt; 2 Packaging Materials Department, National Research Centre, 33 El Bohouth St. (former El Tahrir St.), Dokki, Giza, 12622 Egypt

**Keywords:** Biological techniques, Biotechnology, Chemistry, Materials science, Nanoscience and technology

## Abstract

Green synthesis selenium nanorods (Se-NRs) were produced based on *Aloe vera* leaf extract. The size, morphology, antimicrobial, and activation of Se-NRs for probiotics were analyzed. The Se-NRS was stable with a diameter of 12 and 40 nm, had an antimicrobial effect, and improved probiotics counts. The microcapsules loaded with Green Se-NRS (0, 0.05 or 0.1 mg/100 ml) and probiotics (*Bifidobacterium lactis* and *Lactobacillus rhamnosus*) were designated with efficiency between 95.25 and 97.27% and irregular shapes. Microcapsules were saved probiotics against gastrointestinal juices. The microcapsules were showed a minor inhibition effect against the cell line. Also, microcapsules integrated into stirred yogurt and exanimated for microbiology, chemically, and sensory for 30 days. The probiotics counts, acidity, total solids, and ash values of samples were increased during storage periods without affecting fat and protein contents. The overall acceptability of yogurt with microcapsules containing probiotics and Se-NRs was high without change in body, odor, color, and appearance.

## Introduction

Food fortification is recognized as a necessary and self-sustaining strategy to enhance the quality and quantity of the nutrients in food. Since the high consumption number of dairy products, the fortification of these products with dietary minerals such as selenium (Se) will successfully decrease or prevent diseases related to nutritional deficiencies. Selenium is a trace mineral that was recently demonstrated to have a valuable role in human health or specific population groups. Selenium fortifications could decrease the risk of some diseases such as cardiovascular disease and thyroid disease. Also, selenium plays a valuable role in the prevention of cancer because of its effects on DNA repair and immune systems including its antioxidant properties as well as other mechanisms^1–4^.

Notwithstanding, milk, and dairy products are poor sources of Se, but it is generally present in traditional meals and consumed usually in moderate amounts especially fermented products. So, that the fortification of Se in fermented products like yogurt is an interesting approach to raising the human intake of selenium^5,6^. Moreover, the supplementation of these products must be carefully controlled to ensure the desired level of forficate in the final products and avoid toxicity. The Recommended Dietary Allowances (RDAs)^7^ for selenium, stated in micro-grams are 70 for adult males, 55 for adult females, 65 for pregnant women, and 75 for lactation.

Fermented dairy products have suitable levels of active cultures, namely probiotics. So, using probiotic bacteria in food is positively vital to deliver valuable health properties in the food industry. Probiotic bacteria as Lactobacilli and Bifidobacteria are food constituents that, when consumed (as in a food or a dietary complement), keep or restore useful bacteria to the gastrointestinal tract^8,9^. Some of the health effects of probiotics include preventive effects against diarrhea, lactose intolerance, low cholesterol, and colon cancer, so the viable count should not lease than 10^9^ CFU/g to give the health influence. Also, the probiotic bacteria were grown anaerobically, which is recognized to have negative electro-kinetic potential and able to attract metal ions for the synthesis of metal nanoparticles in oxidizing and reducing conditions^10,11^. Dairy products have long been used as a probiotic carrier as fermented milk, milk powder, ice cream, cheese, and yogurt and documented as functional dairy products^12–17^. But there are some problems related to the efficiency of probiotics in dairy products as a functional food^18^. Mainly, the viability of probiotics was maintained during the storage of dairy products, and after that through the gastrointestinal condition to their site of action (colon).

Microencapsulation procedures can be used to preserve the probiotics viability during processing of food product, storage, and during passing the gastrointestinal tract^15,19^. Calcium alginate (an anionic linear heteropolysaccharide) is a polymer that has been successfully and commonly used for probiotic bacteria encapsulation. The easy application, safety, and cost-effectiveness prepared it one of the most generally used materials for microencapsulation^20,21^. However, it is sensitive to chelating agents and an acidic environment below pH 2, so, made it is limited in some applications. So, other polymers like chitosan, Arabic gum, guar gum, and carrageenan are categorized as food-grade and non-toxic polymers and can be used as the secondary coat over the alginate beads^22–24^. In general, microencapsulation by alginate or alginate with other materials as a double coating using extrusion method is significantly provide greater protection during exposure to simulated gastrointestinal juices^25,26^.

Green synthesis was providing new methods for the preparation of different nanomaterials with good characteristics^27^. Selenium nanoparticles (Se-NPs) are frequently achieved by reduction of sodium selenate in presence of plant extracts^28^. The metabolites from plant extracts denote a well alternate to chemical approaches to achieve the growing request for non-hazardous nanomaterials green production ways comprising plants, carbohydrates, phenolic compounds, flavonoids amines, alcohols, proteins, tannins, saponins, minerals, iron, vitamins, potassium, calcium, and aldehydes are identified to be possible reducing agents of selenium^29^. Plant extracts can act as creators and protectors of the environment when they are appropriately used^30^. Plant extracts are an excellent technique for green synthesis; meanwhile, this technique does not use any toxic chemicals besides having several benefits, including cost-efficiency, sustainability, environmental, and suitability for different applications such as biomedical and pharmaceutical applications^31^.

Many physical, chemical, in addition to green nanomaterials production approaches have been established and employed to fabricate several nanomaterials^32^. While, green synthesis methods are considered eco-friendly, economic, and one-step techniques because of utilizing natural bio-reductants and stabilizers in the fabrication of the nanomaterials^33^. Plants extracts have been extensively used in the preparation of the nanomaterials as compared to the microorganisms; it may be associated with the removal of the cell culturing and isolation^34^. *Aloe vera plant*, as a significant medical plant, contains peel (leaf) and gel, which numerous studies have been displayed that *Aloe vera plant* leaf comprises several vitamins, proteins, polysaccharides, lignin, saponins, sterols, phenolic compounds, and flavonoids that have a vital role in the reduction of the ions to their element and produce their nanomaterials, as well stabilizing of the manufactured nanomaterials^28^. Many factors have affected the characteristics of the prepared nanomaterials, containing temperature, pH, type besides the concentration of the leaf extract, kind and concentration of the precursor's ions, and other functioning factors that are powerfully dependent on the using approaches^27^. Hence, the current work intensive on assessment of the possible use of the *Aloe vera* leaf extract in the preparation of the selenium nanostructures.

The current works aimed to prepare green syntheses Se-NRs based on *Aloe vera plant* leaf extract. The preparation and characterization of the microcapsules containing both Green-Se-NRs and probiotic strains mainly (*B. lactis* and *Lb. rhamnosus*) using the extrusion method were done. Furthermore, the produced yogurts supplemented with functional microcapsules were investigated in terms of chemical, microbiological, and sensory properties during 30 days of storage period. To your knowledge, it is the first time to prepare yogurt with high nutritional value supplemented with functional microencapsulated green sensitized Se-NRs with probiotic strains.

## Materials and methods

### Materials

Sodium selenite (Na_2_SeO_3_), as a precursor of Selenium nanorods (Se-NRs), was purchased from Sigma Aldrich (USA). Green fresh leaves of *Aloe vera* with similar color, shapes, and out of the physical destruction were obtained from a local market (Giza, Egypt). Sodium alginate and guar gum were obtained from Loba Chemie, Pvt Ltd—Mumbai, India. Fresh milk was provided by the Faculty of Agriculture, Cairo University, Egypt.

#### Microbial strains

Microbial strains were collected from the dairy department, National Research Centre as:

Probiotic *Bifidobacterium bifidum* NRRL B-41410, *Bifidobacterium lactis* BB12, *Lactobacillus rhamnosus* NRRL B-442, *Lactobacillus paracasei* NRRL B-4564*, Lactobacillus salivarius* NBIMCC 1589 and *Lactobacillus acidophilus* CH-2.

Starter cultures strains used in stirred yogurt manufacturing: *Lactobacillus bulgaricus Lb*-12 DRI-VAC and *Streptococcus thermophilus* CH-1.

Pathogenic and spoilage strains used in antimicrobial activity test: *Salmonella typhimurium* 14028s, *Staphylococcus aureus* ATCC6538, *Escherichia coli* ATCC 8739, *Yersinia enterocolitica* ATCC23715, *Listeria monocytogenes* 598, *Aspergillus niger* 3858a, and *Aspergillus flavus* B-3357.

### Methods

#### Preparation of* Aloe vera* leaf extracts

*Aloe vera* leaves were washed well using fresh water to remove the existing contaminations on the surface of the leaves and subsequently, those leaves were cut into small pieces then dried in dark at room temperature for 7 days. Via a home miller (MX-GX1521; Panasonic, Tokyo, Japan), 20 g dried powder of *Aloe vera* was provided and added into 500 mL of boiling distilled water for 30 min to acquire the *Aloe vera* leaf extract. Finally, the *Aloe vera* extract was filtered by filter paper (Whatman No. 40), and reserved in the refrigerator.

#### Green syntheses of selenium nanorods (Se-NRs) via plant extracts

The selenium nanorods were prepared by a green method using *Aloe vera *extract as a reducing agent. Firstly, sodium selenite (Na_2_SeO_3_) solution (10 mM) was prepared by adding 0.526 g of Na_2_SeO_3_ into the 200 mL of double distilled water. In the classic production of selenium nanorods, 25 mL of *Aloe vera* extract were mixed with 150 mL of Na_2_SeO_3_ solution. Finally, the prepared solution was placed into the Teflon autoclave, adjusted at 130 °C and 1.5 bar, for 60 min.

All methods were carried out in accordance with relevant guidelines and regulations. https://www.nature.com/srep/author-instructions/submission-guidelines.

#### Characterization of the prepared Se-NRs

The prepared Se nanorods were characterized by dynamic light scattering (DLS), particle size analyzer (NICOMP 380 ZLS, PSS, Santa Barbara, CA, USA), transmission electron microscope ((TEM)) at an accelerating voltage of 80 kV.), with an energy dispersive X-ray analysis (EDAX) analyzer ((JEM-1230; JEOL Ltd., Tokyo, Japan), for the size and morphology analysis of the Se-NRs. XRD analysis was studied using Philips diffractometer (PW 1820 goniometer, PW 1930 generator), stationary thru radiation of Cu Kα (45 kV, 40 mA, with λ = 0.15418 nm ).

#### Antimicrobial activity of synthesis Se nanoparticles (Green-Se-NRs)

The activity of Green-Se-NRs against pathogens that cause food poisoning and spoilage was determined by well diffusion test according to El-Sayed and El-Sayed^35^ using the nutrient agar medium. The tested microbes (around 20 µl of 10^4^ CFU) were sprayed by sterile swab regularly on the surface of medium in plates. The wells with 6 mm were prepared in the medium. Each well was filled with 50 µl of Green-Se-NRs. All plates were incubated for 24 h at 37 °C, after the incubation period, the diameter of the inhibition zone around each well was measured as millimeters.

#### The percent of microbial inhibition rate

To confirm the antimicrobial activity of Se nanoparticles (Green-Se-NRs), the percent of inhibition was achieved by the count of tested microbes according to Safaei et al.^36^ the different tested bacterial strains were activated using tryptone soya broth for 24 h at 37 °C, but the fungi strains were activated using mold extract broth and incubated 30 °C for 48 h. The microbes were individually serially diluted using saline solution to obtain the concentration of 10^5^ CFU/ ml. Then the molted 100 mL of nutrient agar medium was mixed individually with Green-Se-NRs at the concentration 0.01 or 0.1/100 mL. Then, the mixed agar medium was added in the sterilized plates (about 15 ml for each plate) and left the plate’s agar medium to be hard. 100 μL of the tested strains suspension were cultured on the agar medium surface using sterilized swap. For the control, 100 μL of different tested strains suspension was cultured on a pure nutrient agar medium without Green-Se-NRs. All plated were incubated at 37 °C/24 h for bacteria and 30 °C/48 h for fungi. The numbers of colonies were counted and the growth inhibition effect of Green-Se-NRs concentration was determined as the equation:

$$\text{Growth} \;  \text{inhibtion} \; \text{rate}\%=\frac{\mathrm{C}-\mathrm{E}}{\mathrm{C}} \times 100$$

where, C: the mean growth of colonies in the control; E: the mean growth of colonies in the experiments.

#### The suitable Green-Se-NRs concentration for probiotic strains activity

The sustainability of probiotic strains in different Green-Se-NRs concentrations (selected according to a daily intake of Se for humans) was measured by counting colony-forming units per milliliter^37^. The probiotic strains were cultivated in MRS broth at 37 °C for 24 h individually, and then 3% (w/v) of activated strains was transferred to 100 ml of MRS broth supplemented with 0, 0.05, 0.1, or 0.15 mg/100 ml. The flasks of MRS medium were incubated at 37 °C. When reaching the post-log phase, 1 ml from each flask was sampled to prepare serial dilutions by saline solution (0.9% NaCl). Then the suitable dilutions were plated on MRS agar and incubated at 37 °C until the colonies appeared for 48 h. The Green-Se-NRs concentrations in which probiotic strains had the maximum viabilities were used in the next microencapsulation techniques.

#### Microencapsulation of probiotic strains with Green-Se-NRs using extrusion method with double coating

The microencapsulated agents consisted of 3% (w/v) sterilized sodium alginate and 0.2% (w/v) sterilized guar gum. The selected probiotic strains (*B. lactis* and *Lb. rhamnosus*) were grown and incubated in MRS broth under anaerobic conditions at 37 °C for 24 h. The high biomasses of cell pellets were obtained by centrifugation at 6000 rpm, for 15 min at 4 °C^22^. The mixture of 100 ml sodium alginate, 25 ml of probiotic cells pellets and selected Green-Se-NRs concentration (0, 0.05, or 0.1 mg/100 ml sodium alginate) were homogenized using magnetic stirring for 10 min. Then, the mixture was transported into a syringe 5 ml (0.5 mm) and extruded into the hardening solution CaCl_2_, 0.2 M with stirring for 30 min. The microcapsules were washed with distilled water and filtrated. After that, the formed microcapsules were dipped in 200 ml of guar gum solution for 20 min to make the double coating and wash again with distilled water. The filtrated different microcapsules were stored for the manufacturing of stirred yogurt.

##### Microencapsulation efficiency (EE)

One gram of each microcapsule was added in 9 ml of sterile tri-sodium citrate solution (2%, w/v) and vortexes till completely broken microcapsules. The counts of probiotic strains inside the broken microcapsules were detected on MRS agar by pour plate method and incubated anaerobically at 37 °C/48 h^20^. Then, the Encapsulation efficiency (EF) for probiotic strains was calculated like:

$$ {\text{EE }} = {\text{ Log}}_{{{1}0}} {\text{N}}/{\text{Log}}_{{{1}0}} {\text{No }} \times { 1}00 $$

where: N = the total of the probiotic’s cells inside the microcapsules, No = the total of the free probiotic’s cells added to the sodium alginate.

##### Morphology of microcapsules

The scanning electron microscope (SEM) model Quanta 250, a high-resolution field emission gun (HRFEG, Czech) was used to determine the microcapsules morphology after coating gold using a vacuum sputtering coater (Edwards S15).

##### In vitro gastrointestinal system tolerance and microencapsulated probiotics survival

The artificial gastrointestinal solutions used prepared as: for saliva solution, 0.220% gastric mucin was added to distilled water with 0.038 NaCl, 0.021 CaCl_2_, 0.073 K_2_HPO_4_, and 0.111 KCl (w/v %), and the pH was adjusted to 7.00. For gastric solution, 0.3% pepsin was added to distilled water with 0.9 NaCl (w/v), and pH adjusted to 2. For intestinal solution, 1.0% pancreatin was added to the distilled water with 0.3 bile salts, 0.65 NaCl, 0.083 KCl, 0.022 CaCl_2_, 0.138 NaHCO_3_ (w/v %) and the pH was adjusted to 7.0.

Ten g of each microcapsule was added into 100 ml firstly to saliva solution for 5 min. The solution after the time was replaced with a gastric solution for 2 h. This followed by replacing the solution with an intestinal solution for 8 h. One gram of each microcapsule was collected at zero and 5 min from saliva solution as well as after 2 h from gastric solution and after 2, 4, 6, and 8 h from intestine solution intervals of incubation at 37 °C. After collecting the microcapsules for each time point, one gram of the viable cell microcapsules was first diluted (1:10) in 3% tri-sodium citrate to release the cells and after that serially diluted in saline solution.

Then, the suitable diluted was plated by MRS agar medium and incubated for 48 h at 37 °C anaerobically^22^.

The release of probiotics from the microcapsules was determined by Lotfipour et al.^38^. Ten grams of each microcapsule was transferred into 100 ml simulated colonic solution (0.1 M KH_2_PO_4_, pH 7.00), mixed gently, and incubated at 37 °C. At 0, 1, 2, 3, 4, 5, 6, 7, and 8 h time intervals, (1 mL) were taken and viable counts were enumerated with serial dilution by saline. The suitable diluted was plated using MRS agar medium and incubated for 37 °C for 48 h, anaerobically.

#### Anticancer activities of microcapsules loaded with probiotics and Green-Se-NRs (0.1 mg/100 ml sodium alginate)

Cytotoxic activity test was performed on Caco-2 Human colon cancer cell and HepG-2 Human liver cancer cell. The cells were inoculated in Roswell Park Memorial Institute (1640) medium in 96-well microtiter plates at a concentration of 10^3^ cells/ well and then incubated for 37 °C/24 h under 5% CO_2_. The IC50 values of microcapsules were determined after dissolving and prepared concentration (0, 62.5, 125, 250, 500 and 1000 µg/ml) using curve-fitting methods with a statistical analysis program according to the methods described by Amer et al.^39^.

#### Stirred yogurt manufacturing by microcapsules

Fresh cow’s milk was heated at 80 °C for 15 min and cooled to 42 °C^40^. The starter cultures of yogurt (*Lb. bulgaricus* and *S. thermophilus*) were added at the concentration of 2%. Each prepared microcapsules with Green-Se-NRs and probiotic strains were added to divide a portion of the inoculated milk at a concentration as 100 g of each microcapsule that resulted after capsulation methods were added to 500 ml inoculated milk. The concentrations of Green-Se-NRs in the resulting stirred yogurt were 0, 0.05, 0.1 mg/100 ml milk (corresponding to daily intake). The samples were then transferred into plastic cups (100 ml) and incubated at 42 °C for 4 h until coagulation. After that, the resulting yogurt was stirred and the cups were kept at 7 °C for 30 days.

##### Microbiological evolution of the stirred yogurt treatments during storage

The counts of microencapsulated probiotic strains found in samples were determined after the release of cells from the capsules. One gram of each sample was dissolved in 9 ml (3% w/v) tri-sodium citrate and stirred. After that, the other dilutions used normal saline solution. The suitable dilution was plated on MRS agar medium supplemented with vancomycin for determined the *Lb. rhamnosus* counts according to Saccaro et al.^41^. The *B. lactis* counts were determined by MRS agar medium with 2 gm/l sodium propionate and 3 gm/l lithium chloride according to Fayed et al.^42^. The counts of starter cultures were determined after serial dilution of samples by saline solutions and the suitable dilution was plated on an MRS agar medium with pH 5.5 to evaluate the *Lb. bulgaricus* counts and the counts of *S. thermophilus* were evaluated by M17 agar medium according to IDF^43^. The mold and yeast counts were detected by according to Baggerman^44^. Also, the total bacterial counts were determined by nutrient agar medium according to APHA^45^. All microbiological content was evaluated for 30 days of storage weekly intervals and the data was expressed as log CFU/ml.

##### Chemical evaluation of stirred yogurt samples

Stirred yogurt samples supplemented with different microcapsules were analyzed according to AOAC^46^ for evaluated stirred yogurt content of dry matter, protein, fat, and ash. The titratable acidity was assessed as illustrated by Ling^47^. The digital pH meter (Hanna, Germany) was used to evaluate the pH of stirred yogurt samples. All chemical characteristics were determined for 30 days of cold storage at weekly intervals.

##### Sensory evaluation of stirred yogurt

The sensory properties were recorded according to evaluate members in the Dairy department, National Research Centre^48^. The yogurts from the different treatments were judged at fresh and every week of storage.

### Statistical analysis

Statistical Analysis System Users Guide SAS^49^ (SAS Institute, Inc., USA) was used to determine the significant difference among treatments. All data were performed in triplicate and mean values.

## Results and discussion

### The morphology investigation of the prepared green selenium nanorods (Se-NRs)

**Figure 1:**
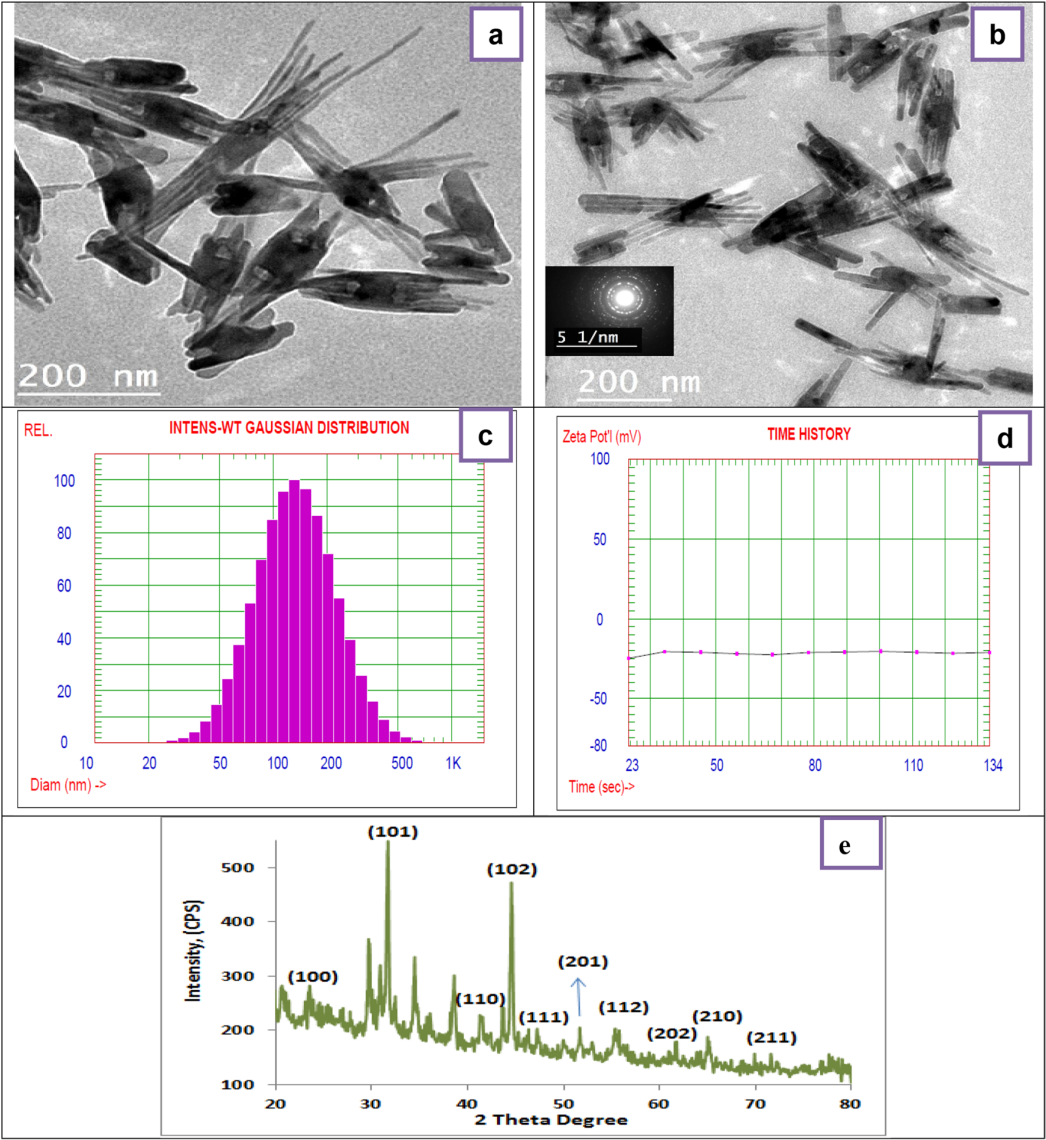
(**a**,**b**) TEM images of Se-NRs produced using green chemistry (different magnifications). , DLS measurement of the prepared Se-NRs prepared via green chemistry, (**c**) particle size, (**d**) zeta potential, (**e**) XRD pattern of Se-NRs prepared via green chemistry.

In order to spread the green technique for the fabrication of inorganic 1D nanostructure in the current work the preparation of Se-NRs from Na_2_SeO_3_ as a precursor using the green method without using any template or surfactant. It is a simple, fast, and low-cost process to produce selenium nanorods (1D nanostructure) on a large scale. The morphology of samples was investigated with TEM. Figure 1a,b displays the TEM investigations of the prepared Se-NRs using green chemistry. Figure 1a,b revealed the sample consisted of Se-NRs with diameters ranging from 12 to 40 nm and lengths of 130 to 230 nm. Moreover, Se nanowires with higher aspect ratios (length to diameter ratio) were also observed in the TEM analysis, as displayed in (Fig. 1a). The detected morphologies of the prepared Se nanostructure by the green method were absolutely nanorods, and no other morphologies were noticed. Furthermore, the SAED pattern (Fig. 1b) of Se-NRs was displayed the diffraction ring pattern indexed as (100), (101), (110), (102), (111), (201), (112) and (202) reflections, demonstrate the creation of selenium nanorods in the hexagonal phase.

### DLS and XRD analysis of the prepared green Se-NRs

The fabrication of selenium nanoparticles was established using laser diffraction (DLS), demonstrating that the particle size distribution achieved through reduction of sodium selenite in the presence of plant extracts as a reducing agent which formed a highly dispersed mixture was maximum 152 nm as shown in (Fig. 1c). Furthermore, the prepared selenium nanorods were very stable, the zeta potential measurement displays negative values, and it was − 21 mV as revealed in (Fig. 1d). The high negative value charge demonstrates the stability of the Se-NRs without forming aggregates; these particles do not convert to black amorphous throughout the storage period.

Figure 1e displays the XRD pattern of typical Se-NRs prepared via green chemistry using Na_2_SeO_3_ as a precursor. The obtained result from Fig. 1e it was revealed that the Se-NRs are a single phase of well-crystallized elemental Se with hexagonal structure. This designates that plant extract performed as a reducing reagent and reduced Na_2_SeO_3_ to elemental Se. Correspondingly, the diffraction peaks at 2θ = 23.4, 29.7, 41.2, 44.5, 46.0, 51.7, 55.8, 61.5, 64.9 and 68.9 were for the (100), (101), (110), (102), (111), (201), (003), (202), (210) and (211), respectively. The reflections of the pure hexagonal phase of selenium nanorods with lattice parameters a = 4.366 Å and c = 4.9536 Å (JCPDS 06-0362). The sharp Bragg reflection was recognized to the occurrence of t-Se nanorods, which is matching with SAED results.

### Antimicrobial activity of Green-Se-NRs

The ability of Green-Se-NRs to a minimum the growth of tested strains was found in (Table S1). The data was indicated that the used Green-Se-NRs concentration (50 µg/each well) had antimicrobial activity more against Gram-negative than Gram-positive strains, additionally had an effect against fungi. The highest antimicrobial activity of Green-Se-NRs was seen in the order of *Y. enterocilitica* (24 mm), followed by. *S. typhimurium *and *A. niger* (22 mm), *E. coli *(21 mm), *A. flavus *(20 mm), *L. monocytogenes *(19 mm), and the lowest inhibition against *S. aureus *(12 mm). These results were confirmed by El-Deeb et al.^50^; Kheradmand et al.^51^ that found the concentration of 100 µg/ml of synthesis Se nanoparticle had antimicrobial activity, but the lowest concentration 2.5–10 μg/ml had no antimicrobial effect on tested strains. Menon et al.^52^ confirmed that synthesized Se nanoparticles own antibacterial activity against Gram-positive and Gram-negative strains, and the highest activity was observed for *Klebsiella* sp. So, the Green-Se-NRs can be further used for numerous pharmaceutical or industrial applications.

### The percent of microbial inhibition rate

The growth inhibition of the tested microbes was investigated (Table 1). The Green-Se-NRs concentrations had antimicrobial effect against tested microbes that able to reduce the colonies counts of microbes when used the two concentrations. At the concentration 0.01/100 mL of Green-Se-NRs, the growth inhibition rate was ranged between 19.54 and 40.46%. When used the concentration 0.1/100 mL of Green-Se-NRs, the growth inhibition rate was increased to range between 39.18 and 64.55%. Also, from the data, the more virulence effect of Green-Se-NRs was observed against Gram-negative bacteria especially *Y. enterocolitica* than Gram-positive bacteria, which the most resistant strain was *S. aureus*. These results were related to the cell wall composition. The cell wall of Gram-positive bacteria was contained peptidoglycan that considerable thicker than Gram-negative bacteria. Nanoparticles were easier to penetrate the cell wall of Gram-negative bacteria and damaged the integrity of the cell membrane^53^. Therefore, it was indicated that Green-Se-NRs exhibited stronger antibacterial properties against Gram-negative bacteria. Moreover, the Green-Se-NRs had bactericidal effect against fungi, where able to reduce the growth of *A. niger* and *A. flavus* to 21.34 and 31.00% at the concentration 0.01/100 mL of Green-Se-NRs, and the growth inhibition was enhanced to record 45.69 and 58.48% with the concentration 0.1/100 mL of Green-Se-NRs, respectively.

**Table 1:** The percent of microbial inhibition rate.

Microbial strains	Control	0.01% Green-Se-NRs	Growth inhibition %	0.1% Green-Se-NRs	Growth inhibition %
*S. typhimurium*	6.518^A^	4.373^B^	32.68^B^	3.086^B^	52.65^C^
*E. coli*	6.607^A^	4.001^B^	39.44^A^	2.903^C^	56.06^B^
*Y. enterocolitica*	6.378^A^	3.799^C^	40.46^A^	2.261^D^	64.55^A^
*L. monocytogenes*	6.029^A^	4.260^B^	29.34^B^	3.149^B^	47.76^D^
*S. aureus*	6.477^A^	5.211^A^	19.54^D^	3.939^A^	39.18^E^
*A. niger*	5.342^B^	4.202^B^	21.34^D^	2.901^C^	45.69^D^
*A. flavus*	5.360^B^	3.698^C^	31.00^C^	2.225^D^	58.48^B^

Means with capital letter superscripts indicate insignificant difference between columns.

### The suitable synthesis (Se-NRs) concentration for probiotic strains activity

The sustainability of probiotic strains in different Green-Se-NRs concentrations (0.05, 0.1, or 0.15 mg/100 ml MRS) was shown in (Fig. S1). The results indicated that different probiotic strains could utilize the small concentration of Green-Se-NRs (0.05 and 0.1 mg/100 ml) found in MRS medium broth without affecting their viability. Moreover, the counts of all tested strains significantly increased than control. The counts of tested strains were decreased when used Green-Se-NRs at the concentration of 0.15 mg/100 ml, but these counts were near to control. Also, the data found that the viable counts of strains *Lb. salivarius,* *Lb. rhamnosus* and *B. lactis* were more than the others, especially at the concentration 0.1 mg/100 ml of Bio-Se-NRs, which the counts of these strains were indicated in ten log cycles. Many Lactobacilli strains can be utilized and bio-transform toxic selenite into non-toxic seleno-amino acids and accumulate inside the cells^54^. Spyridopoulou et al.^55^ observed under an optical microscope that *Lb. casei* ATCC 393 was grown in the presence of NaHSeO_3_.

The count was determined by counting the colonies grown on agar medium and found to be 1.037 × 10^9^ CFU/ml. Also, González-Olivares et al.^56^ detected that the lactic acid bacteria can survive in concentrations over 200 mg/L of Na_2_SeO_3_. Kang et al.^57^ indicated for lactobacilli species grew in the presence of 60 mM selenite, including *Lb. plantarum, Lb. pentosus*, *Lb. fermentum*, and *Lb. rhamnosus* and all these four strains can grow in 100 mM ZnSO_4_-7H_2_O. Moreover, different lactic acid strains can biosynthesize nano elements like zinc oxide and selenium nanoparticles as *Lactobacillus gasseri* was produced ZnO-NPs with a high amount reached of 164 mg/g^58^. So, the utilization of lactobacilli that accumulated with Se-enriched owns unique advantages, including low-cost production and additional probiotic benefits.

Generally, the selective mechanism of Green-Se-NRs against pathogenic microbes and probiotic strains was in-depended on the type of strain and their ability to consume the metal nanoparticles or not. In case of pathogens, the small size and highly surface of Green-Se-NRs was improved the antimicrobial action, which allows a close interaction with bacterial membranes and induce loss of membrane integrity, oxidative stress, and injury to proteins and DNA^59,60^**.** But, in the case of probiotic strains, the probiotic strains could utilize the metal ions easy^61^. Which, two genes SelA and SelD are located in the probiotic strains genome and involved in Se metabolism^62,63^. So, the helpful effect of nanoparticles delivered to the gut microbiota, aiming to reduce the pathogens, by eliminated the growth of harmful microbes and improved beneficial bacteria as probiotics^64,65^.

### Microencapsulation efficiency (EE)

The initial probiotic cells count before microencapsulation was 9.27, 9.55, and 9.79 Log CFU/ml, respectively for M1, M2, and M3 as revealed in (Table 2). There were significant differences in microencapsulation efficiency (EE) of microencapsulation for M1 (without Green-Se-NRs) and other microcapsules (M2 and M3 that contained Green-Se-NRs). The percentage of efficiency after microencapsulation was recorded 95.25, 96.93, and 97.27% for M1, M2, and M3, respectively. So, our results indicated that the efficiency of the microencapsulation process slightly improved by adding Green-Se-NRs with non-significantly differences between microcapsules. This finding may be improved according to that Green-Se-NRs filled the pours found in the network of materials used in the microencapsulation process. Others study by Fayed et al.^22^ demonstrated that addition inulin fabricated into PLGA nanoparticles inside the beads with mixed strains of Bifidobacterium was significantly improved the microencapsulation yield. Also, Liao et al.^66^ found that the *Lb*. *fermentum* L7 cells co-encapsulated yield with oligosaccharides was significantly higher than that of cells encapsulated with alginate alone.

**Table 2:** Microencapsulation efficiency (EE).

Microcapsules type	Initial counts (log CFU/ml)	Entrapped counts (log CFU/g)	EE, (%)
M1	9.27	8.83	95.25 ± 1.36^A^
M2	9.79	9.49	96.93 ± 1.48^A^
M3	9.55	9.29	97.27 ± 1.70^A^

M1: probiotic strains microcapsules, M2: probiotic strains and 0.05 mg Green-Se-NRs microcapsules, M3: probiotic strains and 0.1 mg Green-Se-NRs microcapsules. (mg/100 ml sodium alginate). Data were expressed as mean ± standard error.

### Morphology of microencapsulated strains

Morphological determination for microcapsules by SEM images (Fig. S2A). All microcapsule types were in the same irregular shape forms with the unformed, wrinkled, and the rough surface was observed, could be attributed the high vacuum applied during SEM analysis as also observed by Mahmoud et al.^20^ and Su et al.^67^. Moreover, the wet image for microcapsules (Fig. S2B) was shown by the normal camera, all microcapsules were near in size and shape with round and smooth surface. Not detected differences in the images by the addition of Green-Se-NRs concentration between all microcapsules.

### In vitro gastrointestinal system tolerance and microencapsulated probiotics survival

**Figure 2:**
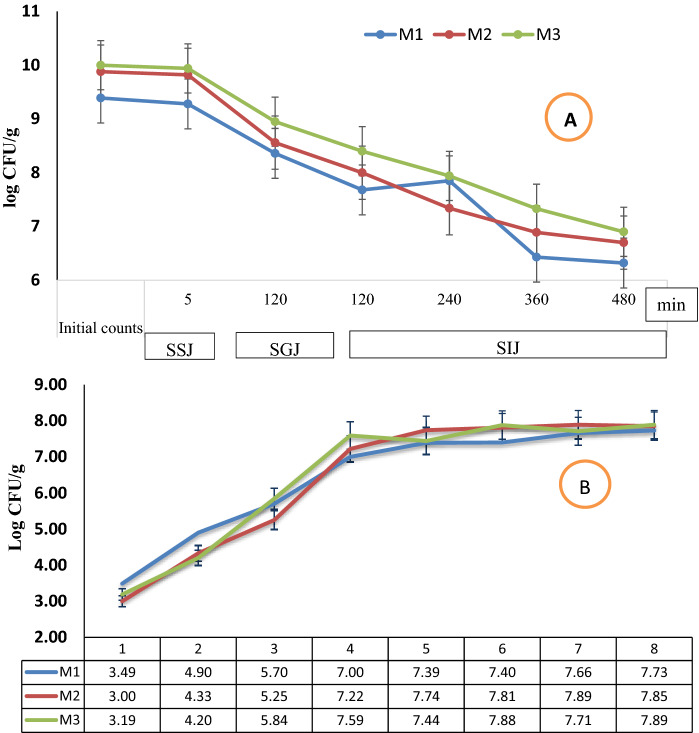
(**A**) Viability of Microencapsulated probiotic strains and Green-Se-NRs (mg/100 ml sodium alginate) in stimulated gastrointestinal system (log CFU/g), (**B**) release of probiotic strains from different microcapsules (Release time, hour). M1: probiotic strains microcapsules; M2: probiotic strains and 0.05 mg Green-Se-NRs microcapsules; M3: probiotic strains and 0.1 mg Green-Se-NRs microcapsules.

The viability of microencapsulated probiotics, when found in the *in-vitro* gastrointestinal system, was recorded in (Fig. 2A). In the first 5 min, all microcapsule types were exposed to stimulated saliva juice, the viability of probiotics inside capsules was relatively found in the same log cycles as initial counts (9.28, 9.82, and 9.94 log CFU/ml, for M1, M2, and M3, respectively). After that, the microcapsules were found in the stomach (Stimulated gastric juice). This is the main point at which the extreme viability counts of probiotics may be lost due to acids and pepsin. The counts for all capsules gradually decreased after replacing the solution with simulated gastric juice (SGJ) for 2 h, which the counts were recorded as 8.36, 8.56, and 8. 95 log CFU/ml for M1, M2, and M3, respectively.

After passage through the SGJ, the microcapsules will enter the small intestine (stimulated intestinal juice), the counts for all capsules were gradually decreased with the time of exposure to SIJ, which the counts reached 6.23, 6.70, and 6.90 logs CFU/ml for M1, M2, and M3, respectively after 8 h. Our data was not indicated significant differences between microcapsule types during the experiment. All the microcapsules can save the survival of the probiotics when passing through the stimulated gastrointestinal system with adequate counts. Moreover, microcapsules of probiotics using double coating materials (alginate and guar gum) with Green-Se-NRs may be added more protections to probiotic strains, as previously recorded by Mahmoud et al.^20^ who found that the survivability of *Lactobacillus plantarum* microencapsulated with alginate combined with other materials was enhanced when found in stimulated gastric and intestinal juices. Also, our results in the same trend with other studies by Malmo et al.^68^; Marefati et al.^69^; Liu et al.^70^; Brinques and Ayub^71^; Chávarri et al.^72^ demonstrated that the microencapsulation process developed survival rate of probiotic cells after GI conditions contact.

It is necessary to confirm that the microencapsulated probiotics are released in simulated colonic solution. The probiotics release counts from the prepared microcapsules was illustrated in (Fig. 2B). According to the obtained results, during the first 1 h incubation in simulated colon solution, the cells initiated released from all the microcapsules to reach the counts between 3.00 and 3.49 logs CFU/g, without significant differences between microcapsule types. During the time, the release from all microcapsules significantly increased, and the maximum counts of probiotics were indicated at the 4 h incubation in stimulated colonic solution. The count's success to reach the maximum, which recorded between 7.00 and 7.59 log CFU/g at 4 h for all microcapsules. After that, the released counts for all prepared microcapsules were constant in the 7 log cycles during 8 h incubation in colonic solution.

It can be concluded that the addition of Green-Se-NRs inside beads with probiotics had no considerable effect on the release of probiotic strains from the prepared microcapsules. Additionally, using guar gum as the second layer material for encapsulated cells may be increased the time of release in stimulated colonic solution. Other studies by Lotfipour et al.^38^ found that the maximum release of *Lb. acidophilus* from alginate and psyllium beads was indicated after 3 h of incubation in colonic solution. Also, Haghshenas et al.^73^ indicated the release of encapsulated *Lb. plantarum* 15HN with the low concentration of alginate could be completely from the beads after 1 h. But, the higher concentrations of alginate significantly reduced the release rate of probiotics and the complete release was observed after 2 h.

### Anticancer activities of probiotics with Green-Se-NRs microcapsules

**Figure 3:**
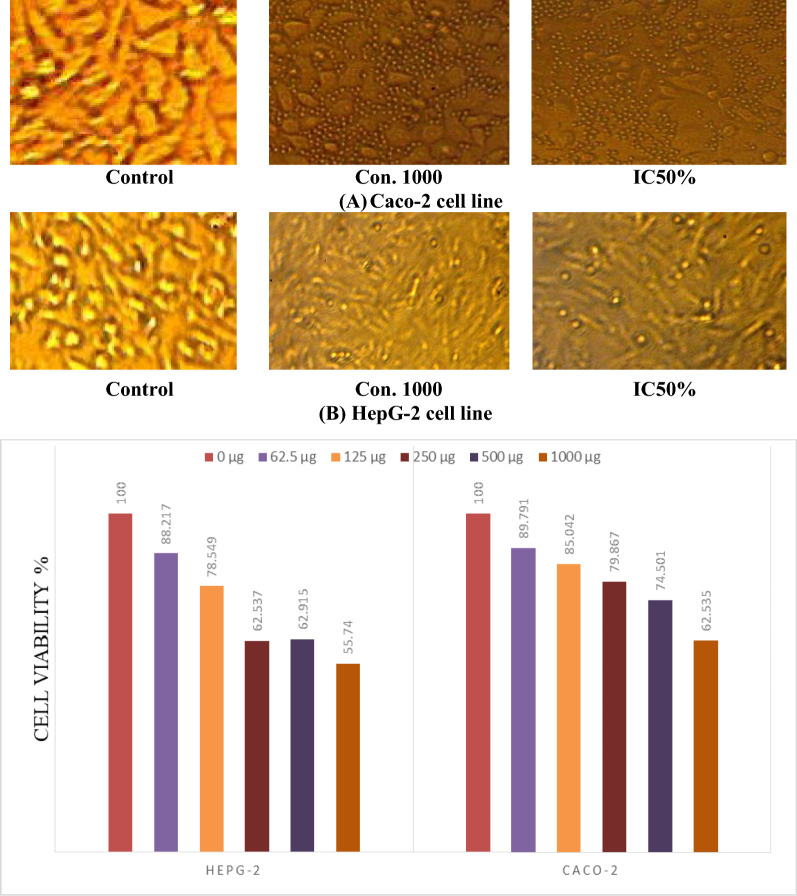
(**A**,**B**) Different cell line viability after 24 h incubation of dissolved microcapsules loaded with probiotics and Green-Se-NRs **(**0.1 mg/100 ml)**.**

The anticancer activity of microcapsules of probiotic cells (*B. lactis* and *L. rhamnosus*) with Green-Se-NRs concentration (0.1 mg/100 ml) was determined by measuring the ability of these microcapsules to inhibit the proliferation of Caco-2 and HepG-2cell lines after dissolving the capsules and preparing different concentration. It can be seen from the data presented in (Fig. 3) the microcapsules were showed a minor inhibition effect against the two types of the cell line. The anti-proliferative effect was increased with the concentration, which the concentration 1000 µg/ml was gained 44. 26 and 37.46% inhibition for HepG-2 and Caco-2 cell lines, respectively. Also, the data was indicated the IC50% against two types of cell lines more than 1000 µg/ml (IC50% was determined as 1260 and 2575% for HepG-2 and Caco-2 cell lines).

On contrary, other results with Faghfoori et al.^74^ found that different Bifidobacterium sp. showed anticancer activity on colorectal cancer cells, and the IC50s after treatment by cell-free supernatants of Bifidobacterium sp. were determined a range between 65 to 80 µg/ml for Caco-2 cells. Abbas and Abou Baker^75^ indicated that biosynthesis of Se-NRs owns potent anticancer against colon, skin, and lung cancers with IC50 10.24, 13.27, and 20.44 μg/ml, respectively.

### Microbiological evolution of the stirred yogurt treatments during storage

**Figure 4:**
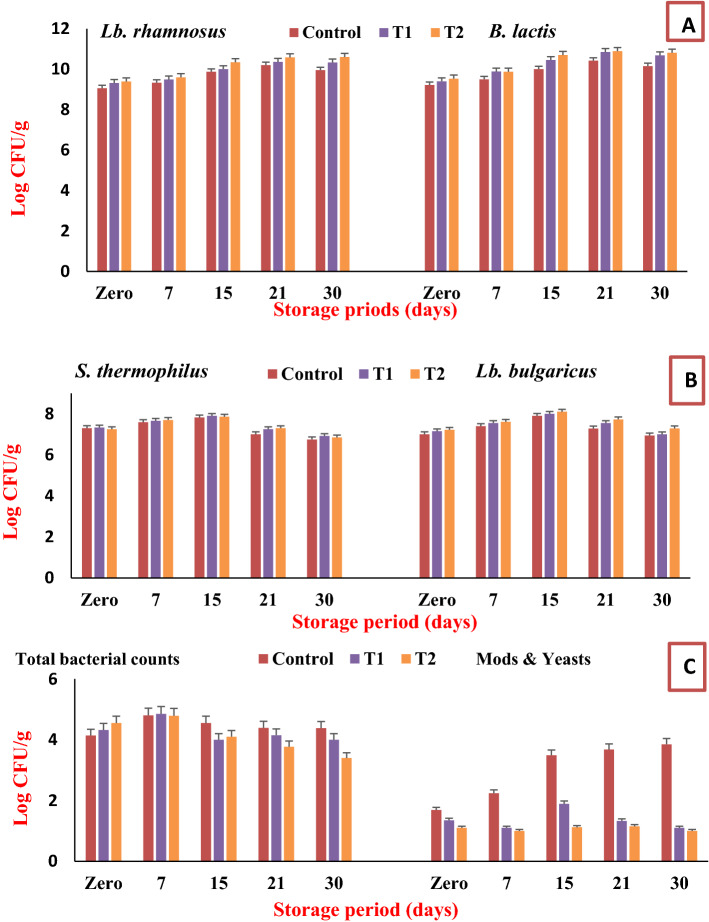
Microbiological characteristics of stirred yogurt fortified with microencapsulated probiotic strains and Green-Se-NRs. Control: yogurt with probiotic strains microcapsules; T1: yogurt with probiotic strains and 0.05 mg/100 ml milk Green-Se-NRs microcapsules; T2: yogurt with probiotic strains and 0.1 mg/100 ml milk Green-Se-NRs microcapsules.

Generally, Selenium (Se) is an important trace element for human health, and the fortification of dairy products with it was improved their nutritional values. Figure 4 displayed the microbiological evaluation of yogurt treatments that fortified with different microcapsules during the storage period. It is clear from the figure that the counts of probiotic strains were significantly enhanced during storage periods (Fig. 4A). The counts of *Lb. rhamnosus* in fresh (1st day) were ranged between 9.03 and 9.39 log CFU/g, after that the counts increased with the storage time to reach between 9.95 and 10.60 log CFU/g on the 30th day. Also, the more counts indicated in T2 that fortified with Green-Se-NRs at concentration 0.1 mg/100 ml milk. The same manner was observed for *B. lactis*, wherein fresh (1st day), the counts were ranged between 9.22 and 9.53 log CFU/g. The counts significantly enhanced during the storage period to reached between 10.15 and 10.81 log CFU/g on the 30th day. Likewise, a slightly significant difference was noticed between treatments, and the more counts boosted in T2. Another factor that affected the viability of probiotics was microencapsulation. The microcapsules form for probiotic strains with Green-Se-NRs was maintained the viable counts during the storage period, as well as, protected cells from the acidity that formed from the metabolic activity of starter cultures in yogurt samples during the storage period^19,20,76,77^.

Generally, the count of viable probiotics on the 30th day was higher than 10^7^ CFU/g, which fulfills the regulation given by the CODEX Alimentarius Commission^78^. Our results confirmed by Gonz’alez-Olivares et al.^56^ found that *Lb. rhamnosus* can gown in the media supplemented with Se and the highest tolerance to concentration 198 mg/l and *Lb. helveticus* IUAMI-70129 had the higher survival (76.50%) with a concentration of Se tolerance (43 mg/l). Alzate et al.^5^ studied the effect of added Se to fermented milk at a concentration ranging from 0.5 to 2.5 mg/g. The data showed an increase in the viability of *B. lactis*, *Lb. bulgaricus*, *Lb. casei,* and *Lb. acidophilus* was witnessed for higher selenium contents (Se 2.5 mg), and the maximum survival rate was noticed at 4 weeks of storage. So that, our work was presented functional yogurt were integrated with microcapsules loaded with probiotics and Se nanoparticles.

Additionally, the starter culture of yogurt treatments was enhanced for 15 days of storage and found after that little decline in the counts around 0.5 log cycle (Fig. 4B). For *S. thermophilus,* at fresh (1st day), the counts were ranged between 7.25 and 7.30 log CFU/g and reached at the end of storage (30th day) between 6.75 and 6.85 logs CFU/g. For *L. bulgaricus*, at fresh (1st day), the counts ranged between 7.00 and 7.22 log CFU/g, and after that at 30th day were recorded between 6.94 and 7.29 log CFU/g. Also, significant differences weren’t detected between treatments, where all treatments were almost to the same extent (in the same log cycles). As well, Yang et al.^37^ indicated that the strains were utilized Se found in the medium and found the accumulation amount of Se by *Lactobacillus bulgaricus* and *Streptococcus thermophilus* reached 12.05 and 11.56 μg/ml, respectively, accompanied by maximum living cells when sodium selenite was 80 μg/ml.

Furthermore, Fig. 4C illustrates that the total bacterial counts in all yogurt treatments ranged between 4.14 and 4.55 logs CFU/g on 1st day. The count persisted in the similar log cycle on the 15th day of storage, where the total bacterial count ranged between 4.10 and 4.52 log CFU/g. At the end of the storage period (30th day), the total bacterial counts decreased to 4.00 and 3.60 log CFU/g for treatments T1 and T2 respectively, but they count for control remained in 4.38 log CFU/g. This decline is due to the effect of Green-Se-NRs that were released from microcapsules during storage and had an antimicrobial effect (established by the antimicrobial test as mentioned before). Se nanoparticle is biologically active and can be used to protect food from unscrupulous microorganisms^37,50,79^.

Also, the antimicrobial effect of Green-Se-NRs was completed by the counts of mold and yeast counts, where was detected the shelf life of the final product (Fig. 4C). Small counts of mold and yeast were detected on the 1st day, where ranged between 1.96 and 1.10 log CFU/g. The count was increased in the control gradually to reach 3.85 logs CFU/g on the 30th day (the end of storage). Nonetheless, the count for T1 and T2 was opposite, which was decreased during storage according to the inhibition effect Green-Se-NRs that released from microcapsules during storage periods. The mold and yeast counts on the 30th day were recorded at 1.10 and 1.00 log CFU/g for T1 and T2, respectively.

### Chemical properties of stirred yogurt fortified with functional microcapsules

Chemical properties of stirred yogurt fortified with functional probiotic microcapsules with or without Green-Se-NRs nanoparticles during storage period at refrigerator temperatures up to four weeks were displayed in (Table 3). Data revealed that there was a significant effect in total solids and ash content of probiotic microcapsules enriched yogurt fortified with both levels of synthesis Green-Se-NRs (T1&T2) compared with control stirred yogurt (free Green-Se-NRs), that control had a lower value of ash and total solids content. This increment is recognized to the presence of Green-Se-NRs in T1 and T2 in the prepared probiotic microcapsules which have a good ability to absorb the water. The Se enrich stirred yogurts have better water holding capacities agreement with Achanta et al.^80^ and Osman et al.^81^. The total solids and ash values of stirred yogurt increased significantly (p < 0.05) through the advanced storage period in all treatments, and this was principally due to the rising more acidity in stirred yogurt samples during storage. El-Shibiny et al.^82^ recognized the increase of dry matter content in yogurt during the long age of storage to acid progress which helps to emit the whey from the curd.

**Table 3:** Chemical composition of stirred yogurt fortified with microencapsulated probiotic strains and Green-Se-NRs.

Parameter	Storage period (days)	Treatments		
		Control	T1	T2
Total solids (%)	Fresh	12.25^Ec^	12.40^Db^	12.54^ Da^
	7	12.65^Dc^	12.93^Cb^	13.08^Ca^
	14	12.71^Cc^	12.96^Cb^	13.13^Ca^
	21	12.84^Bc^	13.05^Bb^	13.20^Ba^
	30	12.96^Ac^	13.12^Ab^	13.28^Aa^
Fat (%)	Fresh	3.10^Ba^	3.10^Ba^	3.10^Ba^
	7	3.10^Ba^	3.20^Ba^	3.20^Ba^
	14	3.20^Aa^	3.20^Ba^	3.20^Ba^
	21	3.20^Aa^	3.30^Aa^	3.30^Aa^
	30	3.30^Aa^	3.40^Aa^	3.40^Aa^
Protein (%)	Fresh	3.93 Da	3.93^ Da^	3.91^ Da^
	7	4.00 ^Ca^	3.99^Ca^	3.99^Ca^
	14	4.20^Ba^	4.18^Ba^	4.17^Ba^
	21	4.24^ABa^	4.23^Aa^	4.22^Aa^
	30	4.28^Aa^	4.26^Aa^	4.25^Aa^
Ash (%)	Fresh	0.91^Cb^	0.95^Cb^	0.99^Ca^
	7	0.95^BCb^	0.97^Cb^	1.08^BCa^
	14	0.98^Bb^	1.00^Cb^	1.13^Ba^
	21	1.00 ^Bc^	1.09^Bb^	1.18^Aa^
	30	1.07^Ac^	1.14^Ab^	1.20^Aa^
Acidity (%)	Fresh	1.17^Ca^	1.16^Ca^	1.16^Ca^
	7	1.21^Ca^	1.19^Cb^	1.18^Cb^
	14	1.25^Ba^	1.23^Bb^	1.21^Bc^
	21	1.27^Ba^	1.25^Bb^	1.24^Bb^
	30	1.32^Aa^	1.30^Ab^	1.28^Ac^
pH	Fresh	4.14^Ab^	4.15^Aa^	4.15^Aa^
	7	4.10^Bb^	4.10^Bb^	4.11^Ba^
	14	4.05^Cb^	4.07^Ca^	4.08^Ca^
	21	4.03^Cc^	4.05^Cb^	4.07^Ca^
	30	4.01^Dc^	4.03^Db^	4.05^ Da^

Means with capital letter superscripts indicate insignificant difference between columns (storage) and means with small letter superscripts indicate insignificant difference between rows (treatment). Control: stirred yogurt with probiotic strains microcapsules; T1: stirred yogurt with probiotic strains and 0.05 mg/100 ml milk Green-Se-NRs microcapsules; T2: stirred yogurt with probiotic strains and 0.1 mg/100 ml milk Green-Se-NRs microcapsules.

The data demonstrated that the addition of both levels of Green-Se-NRs in stirred yogurt fortified with probiotic microcapsules did not affect fat and protein contents compared to the control yogurt, while fat and protein percentage increased significantly during storage periods up to four weeks at refrigerator temperature in all yogurt treatments. These results agree with Abd El-Salam et al.^83^; El-Sayed et al.^84^ who reported that during cold storage there is a substantial increase in protein and fat of cheese curd to the corresponding increase in the total solids of cheese. Table 3 displays that; the acidity percent behaves the opposite approach of pH values.

Furthermore, both storage time and Green-Se-NRs fortification had significant (p < 0.05) effects on both titratable acidity and pH values of stirred yogurt. The acidity and pH values for both Se fortified yogurt and control samples are increased for the acidity values and decrease for pH values, respectively, with increasing cold storage times up to four weeks. On the other hand, the data showed that the acid development decreased slightly but significantly (P < 0.05) with increasing Green-Se-NRs levels in stirred yogurt fortified with probiotic microcapsules, which reached 1.30 and 1.28% for T1 and T2, respectively at the end of storage period. The decline of acidity could be due to the Green-Se-NRs, which may influence the growth of lactic acid bacteria. Similar results were observed by Zommara and Prokisch^85^; Osman et al.^81^ who confirmed that there is a decrease in acidity values of stirred yogurt fortified with Green-Se-NRs.

### Sensory properties of the supplemented stirred yogurt treatments during storage

Figure S3 presented the overall acceptability scores of control and yogurt supplemented with different concentrations of Green-Se-NRs during the storage period. The storage period significantly (p < 0.05) affected the overall acceptability that the stirred yogurt supplemented with M2 and M3 (T1&T2) were quite good and recorded a higher score than control yogurt supplemented with M1 exclusively after 21 days of cold-stored. This can be attributed to appear of the mold and the yeast in the surface of yogurt supplemented with M1, contrary to the yogurt supplemented with M2 and M3, which have a good inhibition effect against the mold and the yeast because it's content of Green-Se-NRs, that had strong antimicrobial activity as mentioned in part 3.1. No significant variance was noticed in sensory properties that include body & texture, odor, color, and appearance of yogurts fortified with M2 and M3, than those of control yogurt supplemented with M1 during the storage period. Similar results were observed by Achanta et al.^80^ who found that yogurts supplemented with small amounts of mineral had no significant difference in sensory properties scores compared with control yogurt.

## Conclusion

In this work, the green technique was used to produce Se-NRs using *Aloe Vera* leaf extract. The produced Green Se-NRs were more stable, nanorods with diameters between 12 and 40 nm. Moreover, it had antimicrobial activity more against Gram-negative than Gram-positive strains and the use of low concentrations (0.05 and 0.1 mg/100 ml) have the ability to activated different probiotic strains. So, our work also designated functional microcapsules loaded with probiotic strains and synthesis Green Se-NRs at ratios (0, 0.05, or 0.1 mg/100 ml) using double coating and extrusion method. These microcapsules gained efficiency ranging between 95.25 and 97.27% with irregular and rough surfaces. All the microcapsules have the same behavior for saving the probiotics when passing through the stimulated gastrointestinal system. Also, the maximum release probiotics counts were indicated at the 4 h in stimulated colonic solution. The ability of microcapsules to inhibit the proliferation of Caco-2 and HepG-2 cell lines after the dissolution was evaluated and found that the concentration of 1000 µg/ml was gained 44. 26 and 37.46% inhibition for HepG-2 and Caco-2 cell lines, respectively. Also, the study produced stirred yogurt fortified with functional microcapsules to deliver the required daily need of selenium and improved the human microbiota by adding probiotics. The results found more probiotic counts, high acidity, total solids, and ash content. The overall acceptability of stirred yogurt fortified with microencapsulated probiotics with both levels of Green-Se-NRs gained a higher score than control yogurt during the storage period, without significant differences for body, texture, odor, color, and appearance.

## Supplementary Information

Supplementary Information.

## Data Availability

All data generated or analyzed during this study are included in this published article (and its supplementary information files) and are available from the corresponding author on reasonable request.
