# Supplementary material for: Designated functional microcapsules loaded with green synthesis selenium nanorods and probiotics for enhancing stirred yogurt
Source: Sci Rep. 2022 Aug 30;12:14751. doi: 10.1038/s41598-022-18781-w (PMC9427739; doi:10.1038/s41598-022-18781-w)
Supplement: Supplementary file 1 — Supplementary Information. [file 41598_2022_18781_MOESM1_ESM.docx]

**Designated functional microcapsules loaded with green synthesis selenium nanorods and probiotics for enhancing stirred yogurt**

**Hoda S. El-Sayed ^a^, Samah M. El-Sayed ^a^*, and Ahmed M. Youssef^b^***

**^a^** Dairy Science Department, National Research Centre, 33 El Bohouth St. (former El Tahrir St.), Dokki, Giza P.O. 12622, Egypt

^b^ Packaging Materials Department, National Research Centre, 33 El Bohouth St. (former El Tahrir st.), Dokki, Giza, Egypt, P.O. 12622

**Supplementary data**

|  |
| --- |

**Figure S1**: Viability of probiotic strains (Log CFU/ml) in the presence of Green-Se-NRs concentrations (mg/100 ml medium).

| **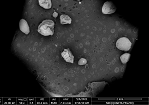M1** | **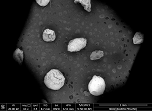M2** | **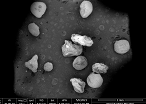M3** |
| --- | --- | --- |
| **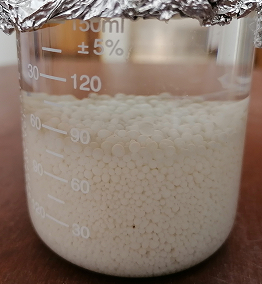M1** | **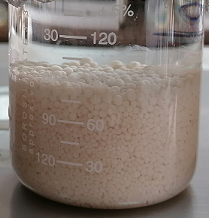**  **M2** | **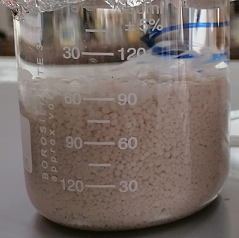M3** |

**Figure S2: A)** SEM images for microcapsules, **B**) Wet image for microcapsules by the normal camera, M1: probiotic strains microcapsules; M2: probiotic strains and 0.05mg Green-Se-NRs microcapsules; M3: probiotic strains and 0.1mg of green-Se-NRs microcapsules

**Figure S3**: Overall acceptability scores of stirred yogurt fortified with different microcapsules during 30 day of storage period. Control: stirred yogurt with probiotic strains microcapsules; T1: stirred yogurt with probiotic strains and 0.05mg/100ml milk Green-Se-NRs microcapsules; T2: stirred yogurt with probiotic strains and 0.1mg/100ml milk Green-Se-NRs microcapsules.

**Table S1:** Antimicrobial activity of synthesis Se nanoparticles (Green -Se-NRs)

| Green -Se NPs (50 µg in each well) | | | | | | | |
| --- | --- | --- | --- | --- | --- | --- | --- |
| Tested strains (mm) | | | | | | | |
| *S.*  *aureus* | ***E.***  ***coli*** | ***S. typhimurium*** | ***L. monocytogenes*** | ***Y. enterocolitica*** | | ***A.***  ***flavus*** | ***A.***  ***niger*** |
| 12^C^ | 21^A^ | 22^A^ | 19^B^ | 24^A^ | 20^B^ | | 22^A^ |
| 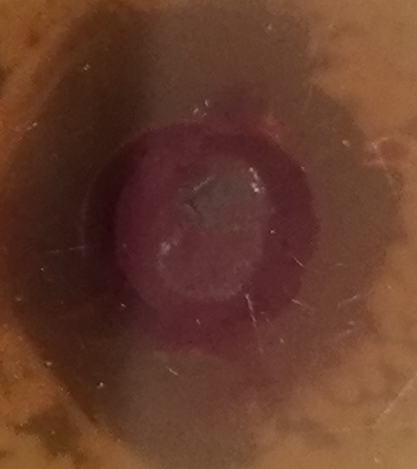 | 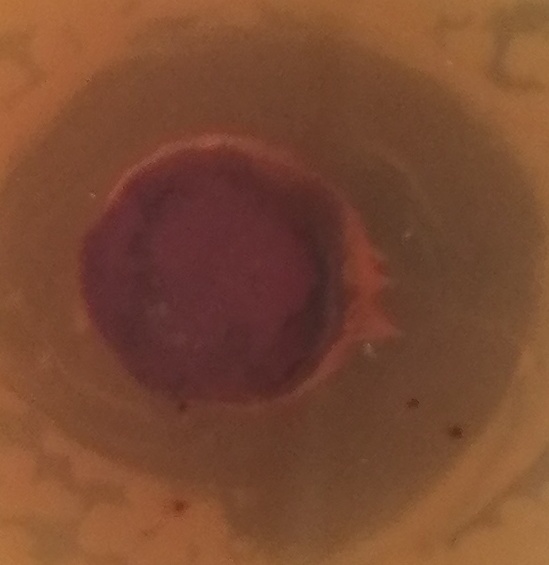 | 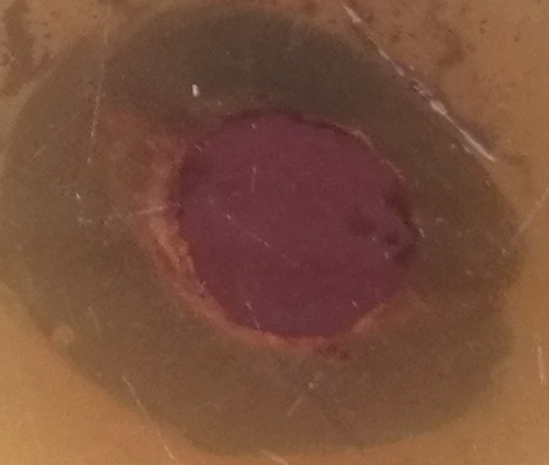 | 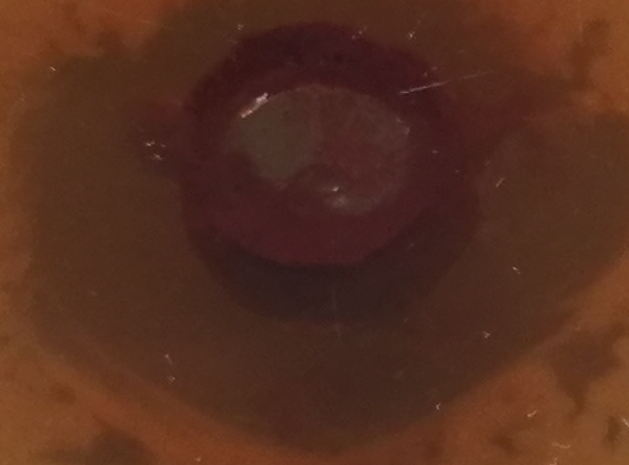 | 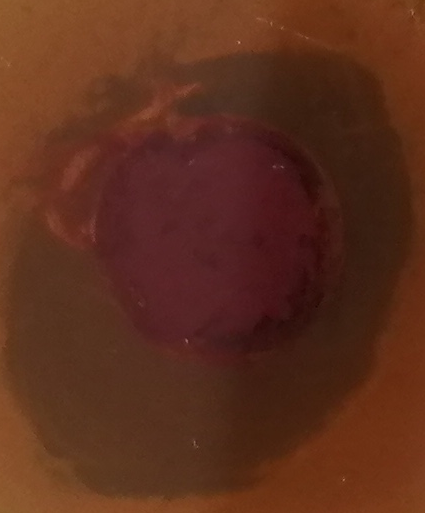 | 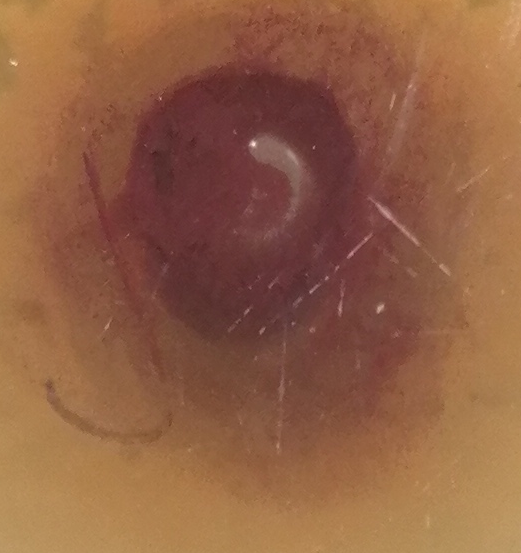 | | 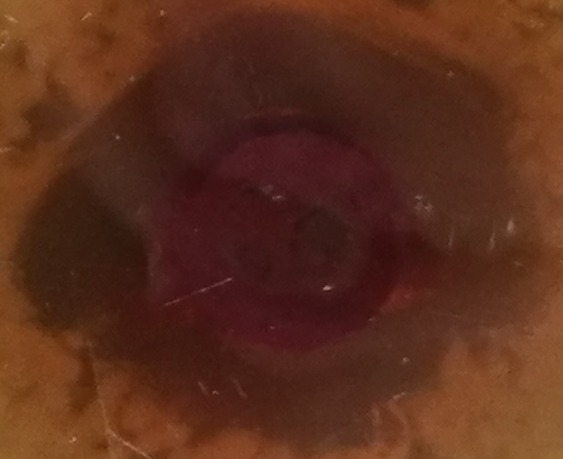 |
